# A Learning Analytics Framework to Analyze Corporal Postures in Students Presentations

**DOI:** 10.3390/s21041525

**Published:** 2021-02-22

**Authors:** Felipe Vieira, Cristian Cechinel, Vinicius Ramos, Fabián Riquelme, Rene Noel, Rodolfo Villarroel, Hector Cornide-Reyes, Roberto Munoz

**Affiliations:** 1 Centro de Ciências, Tecnologias e Saúde, Universidade Federal de Santa Catarina, Araranguá 88906072, Brazil; cristian.cechinel@ufsc.br (C.C.); v.ramos@ufsc.br (V.R.); 2 Escuela de Ingeniería Informática, Universidad de Valparaíso, Valparaíso 2362735, Chile; fabian.riquelme@uv.cl (F.R.); rene.noel@uv.cl (R.N.); roberto.munoz@uv.cl (R.M.); 3 Escuela de Ingeniería Informática, Pontificia Universidad Católica de Valparaíso, Valparaíso 2362807, Chile; rodolfo.villarroel@pucv.cl; 4 Departamento de Ingeniería Informática y Ciencias de la Computación, Universidad de Atacama, Copiapó 1531772, Chile; hector.cornide@uda.cl

**Keywords:** multimodal learning analytics, oral presentations, educational data mining, sequential pattern mining

## Abstract

Communicating in social and public environments are considered professional skills that can strongly influence career development. Therefore, it is important to proper train and evaluate students in this kind of abilities so that they can better interact in their professional relationships, during the resolution of problems, negotiations and conflict management. This is a complex problem as it involves corporal analysis and the assessment of aspects that until recently were almost impossible to quantitatively measure. Nowadays, a number of new technologies and sensors have being developed for the capture of different kinds of contextual and personal information, but these technologies were not yet fully integrated inside learning settings. In this context, this paper presents a framework to facilitate the analysis and detection of patterns of students in oral presentations. Four steps are proposed for the given framework: *Data collection*, *Statistical Analysis*, *Clustering*, and *Sequential Pattern Mining*. Data Collection step is responsible for the collection of students interactions during presentations and the arrangement of data for further analysis. Statistical Analysis provides a general understanding of the data collected by showing the differences and similarities of the presentations along the semester. The Clustering stage segments students into groups according to well-defined attributes helping to observe different corporal patterns of the students. Finally, Sequential Pattern Mining step complements the previous stages allowing the identification of sequential patterns of postures in the different groups. The framework was tested in a case study with data collected from 222 freshman students of Computer Engineering (CE) course at three different times during two different years. The analysis made it possible to segment the presenters into three distinct groups according to their corporal postures. The statistical analysis helped to assess how the postures of the students evolved throughout each year. The sequential pattern mining provided a complementary perspective for data evaluation and helped to observe the most frequent postural sequences of the students. Results show the framework could be used as a guidance to provide students automated feedback throughout their presentations and can serve as background information for future comparisons of students presentations from different undergraduate courses.

## 1. Introduction

Verbal communication is the use of sounds and words to express yourself and is one of the main forms of social interaction. Nevertheless, non-verbal communication, such as facial expression, and body movement, can communicate much more about what people are thinking or feeling than words [1]. Conservative research estimations point that non-verbal signal represents more than 60% in inter-personal communication [2]. Dell Hymes [3] says that only words and sounds are not enough to pass the information to the listeners.

Communicating in social and public environments can influence career development, build relationships, solve problems and conflicts, or even deal with negotiations [4]. Soft skills characterize how a person interacts in his or her relationships with others, such as public speech or communication abilities, and they are very important to any individual for career development [5].

A successful public speech is associated with different skills and factors of the presenter, such as his/her voice, intonation, facial expressions, head position, hands, and body gestures [6]. The ability to speak and present in public can be significantly improved through intensive training [7].

In the 2010 decade, many studies grabbed the attention of the researchers to learn about the students’ gestures. Blikstein and Worsley [8] say that Multimodal Learning Analytics (MMLA) “could offer new insights into learning, especially when students have the opportunity to generate unique, personalized artifacts, such as computer programs, robots, and solutions engineering challenges.” [8]. Extracting, processing, and analyzing audio and video data are part of the MMLA field.

Considering the social nature of the learning process, the application of MMLA for discovering nonverbal attributes such as speech, hands position, and head movement of the students is a promising (but challenging) area, that has been explored in other fields such as the detection of emergent leaders [9].

In an academic environment, the identification of such attributes is relevant to get some insights and information about students’ knowledge, misconceptions, and problem-solving when learning new concepts [10], and to give feedback to them. Feedback is essential for students to achieve their goals [11], and its role in the learning process is to confirm or propose changes in their knowledge [12].

In this context, the main goal of this work is to present a conceptual framework to facilitate the analysis and to detect patterns in oral student presentations. Imenda [13] differentiates conceptual from a theoretical framework, where the former is used by the researcher when it is not possible to explain its research question entirely by a theory presented in previous work. In this case, the researcher must summarize the theory and empirical information found in the literature about a specific problem. This synthesis is called Conceptual Framework, which is an integrated way of looking at a problem [14]. Opportunely, Worsley [15] suggests that the use of a framework can assist in creating standards to analyze data in a variety of contexts, clarifying their objectives and research goals.

The remainder of this paper continues as follows. Section 2 summarizes the work related to different MMLA techniques for data collection and analysis of non-verbal communication. Section 3 presents the different components of the learning analytics framework to analyze corporal postures in student presentations. Section 4 details the experimental design for the case study. Section 5 presents the results obtained for the application of each step of the process considered by the framework. Finally, Section 6 discusses the main results obtained, and Section 7 presents the main conclusions of this work.

## 2. Related Work

The use of MMLA in the academic environment is a relatively new approach. In this section, we present related research to this paper considering two main topics: existing experiences in posture and nonverbal attributes detection using MMLA, and the use of frameworks as a methodological approach to tackle specific concerns in MMLA.

In [16] the authors propose a tool, called Lelikëlen, to visualize and classify a set of 10 body postures using a Microsoft Kinect sensor. The tool was tested and validated according to precision and usability. The authors successfully discriminate similar presentation topics and on similar complexity levels for 45 students. The authors also argue that this tool could be used to give instantaneous feedback to the teacher and the students during oral presentations.

In [10] some findings are presented about how to use Microsoft Kinect sensors data and interaction log data to extract meaningful predictors for student learning. The authors used a Support Vector Machine (SVM) clustering algorithm to predict the students’ learning gain with very high accuracy. Closely, using data gathered from Microsoft Kinect and speech, some analyses have shown a positive correlation between movement and gestures with collaboration and learning gains [17]. Likewise, in [10] the authors use clustering algorithms to relate body gestures with learning gains.

In an academic environment, in [18] the authors recorded practical laboratory classrooms with different cameras and angles. The work explains and predicts high-level constructs such as students’ attention and engagement. In the end, the paper describes the setting, hardware, and software needed to replicate the analytical approach.

In [19], the authors focus on automatic feedback for virtual reality and face-to-face student presentations. Their results show that students using virtual reality perceived the feedback as detailed, and in a face-to-face environment, students perceived the feedback as constructive. However, the authors warn that the research can not be generalized because of the small sample.

In the context of face-to-face presentations, both clustering algorithms and inferential analysis were used in [20] to identify body patterns in student presentations. The authors collected data with a Kinect, and 12 body patterns were found in three different presentation times. In both case studies analyzed, the clustering shows three different clusters, and the analysis points to a convergent number of attributes at the end of the third presentation time.

Although the above initiatives provide a valuable basis for the application of MMLA for specific goals and tasks, extracting nonverbal attributes through is still a technical and methodological challenge. Regarding the later, conceptual frameworks have emerged as a way to systematically approach to the different tasks involved in data collection, processing, analysis, and visualization, in a variety of domains such as collaborative problem-solving [21], class engagement [22], and inter-stakeholder communication [23].

In [21], the authors present a framework that uses non-verbal indices of students’ physical interactivity to analyze problem-solving. The results show an original way to identify different behaviors in collaborative problem resolution.

In an effort to enhance the communicative power of the MMLA in an academic environment, in [24] the authors take some aspects of the data storytelling (DS) to study how visual elements contribute to the DS and how effective is this combination of MMLA and DS. The authors propose a conceptual model that applies elements of the DS in some dashboard aspects. Results show that the prescriptive title, text labels, and shaded areas are elements that add contextual information for teachers.

Based on MMLA applied to physical/motor tasks, the Distributed Cognition theory, and concepts of the internet of things, in [25] the authors provide a theoretical perspective for bringing LA into physical spaces. The study presents prototypes that help teachers and students in collaborative learning, dance education, and healthcare training.

In a study to understand how visual attention can be used to guide the students in a complex animation about the cardiovascular system [26], authors used the Cognitive Load theory and MMLA, registering eye movement, to understand and enhance learning. The results show that students spend greater and more frequent attention in cued parts of the object, but no effects of cueing on visual search and cognitive load.

The computer-supported collaborative-inquiry-learning (CSCiL) environments are used frequently in graduate courses to develop their students’ skills in collaboration, problem-solving, and critical thinking. In [27] presented a study that analyzes the chat message of 144 students of two-year colleges. The authors used transition-rate analysis, entropy-analysis, and sequential pattern mining. The results show, on the one hand, that students in groups that accomplished the tasks were more likely to help each other before moving to the next step. On the other hand, students in groups that did not accomplish the proposed tasks were more likely to regulate the process without reaching a shared understanding.

For [28], MMLA is a way to improve the core skills of students. The authors present an exploratory study that analyzes the collaboration and communication of students in a Software Engineering class of agile practices using Lego4scrum. The study used social network analysis and statistical techniques to identify collaborative and non-collaborative groups from the collected data. The results, as stated by the authors, “offer considerable feasibilities to support the process of skills development in students.”

In the light of the previous works, there are clear advances in MMLA towards posture and nonverbal attributes detection, however these are individual and isolated initiatives, focused on specific learning activities and outcomes. On the other hand, conceptual frameworks are powerful artefacts to guide the MMLA process (commonly with tool support), but there are no initiatives that could be exploited for the goal of our study, regarding students presentations.

## 3. Conceptual Framework Proposal

**Figure 1:**
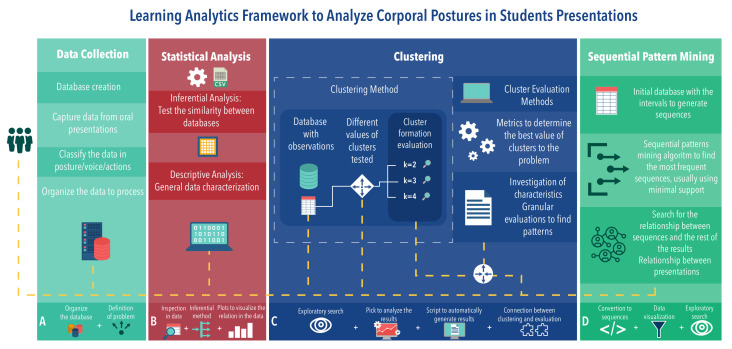
Conceptual framework proposal.

In this work, a conceptual framework for the evaluation of corporal posture patterns in oral presentations is proposed. Figure 1 presents a general outline of the framework proposed. The upper scheme illustrates the flow and some of the steps and processes to be performed on the data. The lower scheme shows different blocks (differentiated by color) representing the processes that must be done in the upper scheme to obtain the results.

This framework can be applied during different periods, for example, over an academic semester. The framework can compare different years and presentations at different times of the semester. The framework uses different variables as a way to externalize and highlight patterns that students have during presentations. It is also possible to group students in the same cluster to facilitate the evaluation of the attributes of said cluster.

The framework has four main blocks (see Figure 1) providing interconnected results. Each block encapsulates the methods necessary to understand the data. In general, the whole process of data analysis follows a flow given by the dashed white dots. However, in several steps, such as setting up the database, it is necessary to return one step or more, since the data can and often contain noise that must be removed or unforeseen errors that can influence the final result.

Briefly speaking, the four blocks can be described as follows—Block A, called Data Collection, indicates how data collection and preprocessing should be done. The success of all other processes depends on the quality of the data acquired in this block. Block B, Statistical Analysis, presents a way to obtain relationships between the years and any number of presentations. With different databases in hand, it is possible to generate relationships among them through statistical methods. Block C, Clustering, aims to segment observations in clusters with well-defined attributes. These results should indicate behaviors that are difficult to visualize without segmenting the observations. Continuing with the intention of displaying student behaviors, Block D, Sequential Pattern Mining, should present interesting sequence patterns, helping educators characterize the presentations, where interesting patterns mean, in this case, a sequence of postures and gestures that could indicate a set of important actions in presentations.

The smaller blocks in the bottom of Figure 1 represent smaller processes that must be performed when using the framework. Each block, individually, represents the steps, or processes, that the data went through. Therefore, each block has its own set of individual results. As it is an exploratory search, these individual results of the blocks must be evaluated and related to the previous results. This relationship can avoid rework when advancing steps in the framework. Table 1 presents a more general explanation, with the inputs of the processes, the processes activities, and the output of each block. The following subsections describe in detail how each block works.

### 3.1. Data Collection (Block A)

The initial data collection and processing process should be considered one of the most relevant stages of the problem since the problem definition is focused here. In this stage, presentations should be collected, defining the data types to be collected. Then, the data already collected should be classified into postures/gestures/actions/voices to identify what the student did during the presentations. The data quality and, therefore, the ability to represent student presentations, will define the problem outcome and the discovery of patterns.

In this step, the data are organized so that several subsequent analyses can be made. The data must be arranged in a way that preserves certain aspects of the presentations. For example, consider the sequences of postures collected from a presentation, represented over time, in instants or intervals. In this case, for further analysis, it may be relevant to know which students made the presentation. Therefore, in addition to the postures, the identifiers of the different students who performed them must also be preserved. This database must contain, for example, the times of each student in each of the attributes of the problem. In this way, the student’s behavior can be tracked in terms of the amount of time in a given attribute and also in a chronological record of what the student did, given by the time interval in each attribute.

The database containing the observations for the rest of the analysis of the results must also be generated. Observations that could be, for example, students with their respective times or other data representing the problem in each of the collected attributes. It is important to note that a larger amount of data (collected attributes) can allow making connections with results between the blocks and methods, which by their own characteristics are different. Data such as the student grades in their presentations or their used slides can also be useful for further analysis.

### 3.2. Statistical Analysis (Block B)

The statistical analysis stage is the first part of the framework’s results that can identify some patterns in data. In this step, an analysis of descriptive statistics is performed. The main objective of this descriptive analysis is to understand the data in general. Data dispersion measures can be used together with charts to show some relevant data behavior.

Besides, an analysis of inferential statistics is also performed, by testing two or more different populations (e.g., the presentations) concerning statistical “similarity”. The test can indicate statistical similarity between presentations and years of evaluation. Or even point out a statistical difference. In both cases, they are important results to understand the students’ learning process. These results can be used to define teaching strategies based on the results of different years of presentations.

The comparison can be, for example, between different years of assessment of the same course, or it can be between different courses of the same year. This setting will depend on how the data was collected. It is noteworthy to pay attention to normality tests before the inferential test, in order to confirm the type of distribution of the presentations.

### 3.3. Clustering (Block C)

Following the dashed line, we arrive at block C, where clustering techniques from unsupervised machine learning are used. One of the objectives of this approach is to segment students into different groups with well-defined attributes for a granular assessment of behaviors. Thus we can differentiate between presentations and find and relate results.

It is difficult to identify the number of groups in a clustering algorithm. In this framework, it is suggested to test different cluster values for the problem in order to state what better represents the data. Clustering assessment methods (e.g., silhouettes [29]) should be used to identify the best number of groups within the presentations.

An exploratory search on the results of the clustering algorithm must be performed. This search should focus on discovering attributes and behaviors in the results that characterize the groups formed by the algorithm. In general, clustering algorithms use some rules to form groups. This discovery can be related to other results, such as the inferences made in the previous step.

The exploratory search is part of the entire process of the framework. However, here it stands out since the formation of clusters contains information on the students’ behavior. Data visualization can clarify patterns that are difficult to observe when looking at a table, for example. This search may be for apparent traits in the formed groups, as a certain predominance of some attributes in some formed clusters.

### 3.4. Sequential Pattern Mining (Block D)

Sequential Pattern Mining appears as an alternative and a complement to the results found in previous analyses. If there is a sequential base with the intervals for each attribute that the student passed, then that base can be used to find sequential patterns in the data.

Thus, the algorithm returns the most present sequences, given a minimum support value. These sequences can be an interesting way to assess student behavior throughout the presentation. For instance, it can indicate a student with lesser participation behavior during the presentation.

Finally, in addition to the sequences, this step should allow the sequences to be visualized in a different way than in the written form. This should be done to facilitate the comprehension of the results. Still, it is importance of having other types of data such as text notes, since they can help to explain better the sequences found.

## 4. Experimental Design

### 4.1. Learning Environment for Presentations

**Figure 2:**
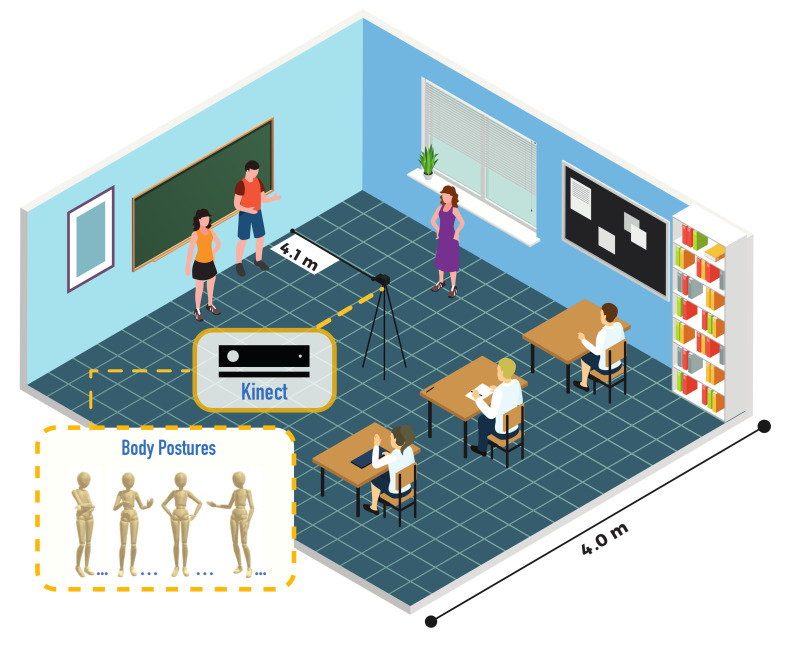
Learning Environment based on [20].

The course “Introduction to Engineering” was offered to students of Computer Engineering at the University of Valparaíso, Chile, during the years 2017 (year 1) and 2018 (year 2). In the course, students made three presentations in groups on three topics previously defined. The learning environment configuration scheme is illustrated in Figure 2.

### 4.2. Lelikëlen Tool

During the course, students must make three oral presentations. These presentations cover the topics of web development, microcontrollers, and databases. The dates of the presentations are about a month apart. Each presentation was made by two students, lasting a maximum of 5 min per pair. The PowerPoint slides, provided by each group and projected on the blackboard, had all the information required to make the students’ presentations. The capturing process of all the performances was taken by the Microsoft Kinect software/hardware and the Lelikëlen application [16]. Also, the methodology was approved by the Ethics Committee of the Faculty of Medicine (University of Valparaíso).

**Figure 3:**
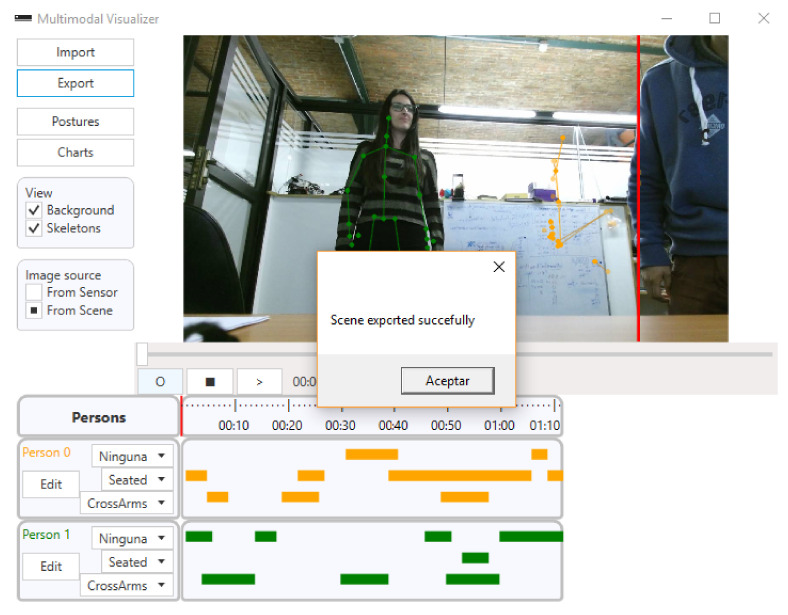
Screenshot of Lelikëlen application showing the postures detected for two people.

The Lelikëlen software was used for data collection and processing (see Figure 3). The software allows us to detect, classify 10 predefined postures, store, and view body postures of recorded people. Lelikëlen has options for adding custom postures, exporting and importing scenes, and view the detected postures along with a timeline. Finally, Lelikëlen also allows exporting the data to be viewed using other data mining tools.

## 5. Results

### 5.1. Data Collection

Based on what was presented in Section 4, the data collected from the presentations are shown in Table 2. The presentations were structured and carried out under equivalent conditions. The idea is to maximize the number of attributes and ensure a higher quality of the data collected, thus facilitating the identification of patterns. Furthermore, the performance of machine learning algorithms depends on the quality of the data collected. As we mentioned before, the data were classified based on the Kinect skeleton models, according to the previous literature [16,30,31,32].

In the educational context of the presentations, the data from the proposed case study are the students’ actions throughout the presentation. The result of this collection in practical terms for the framework may be the diversification of the analyses used in this data set. A total of six presentations were collected, three presentations for each year (year 1 and year 2), as can be seen in Table 3. Due to the configuration of presentations, they can be compared per each year or even together for both years. These comparisons are evaluated in the following sections as we move forward in the framework blocks.

### 5.2. Statistical Analysis (Extraction)

Statistical analysis using descriptive and inferential methods can return some varied results. Descriptive analysis, as the name suggests, can describe and indicate certain trends in the data, through visual methods and data dispersion measures. Consequently, it can describe students’ behavior and part of the general learning environment. Otherwise, inferential statistical analysis projects another perspective about students’ presentations. It can indicate relationships between the attributes of each year, and also a comparison between the attributes of both years, making it possible to state statistically significant similarities and differences between each of the attributes.

**Figure 4:**
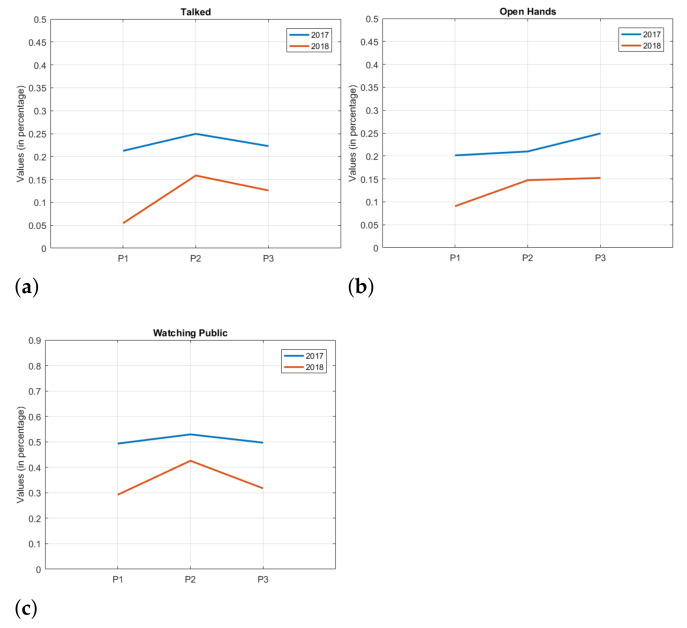
Trend charts comparing years. (**a**) Talked, (**b**) OpenHands, (**c**) Watching public.

In this sense, Figure 4 shows similarities and differences, depending on the figure, between the presentations of year 1 and year 2. P1, P2 and P3 on the horizontal axis represent the presentation periods (see Table 3), and the vertical axis represents the mean values of the attributes, that is, the average percentage of time that the students maintained that attribute in each presentation period. In general, it can be seen that the behavior of presentations between the years are similar for most of the attributes, with a higher attribute value for the year 1 compared to year 2.

From an educational point of view, it is interesting to note that these data allow us to observe certain trends among some attributes. For the attributes *Talked*, *Cross Arms*, and *Watching Public*, for example (Figure 4a represents this trend), it can be seen that when a value falls in one of the years, the other year presents a similar behavior, albeit at a different intensity. The above shows a certain trend and similarity in the behavior of the attributes from one year to another.

On the other hand, *Downside* and *Open Hands* attributes (see Figure 4b) present a different behavior throughout the years.

It is also possible to observe attributes that, at some presentation period, obtain a similar trend, but diverge elsewhere during the semester, such as in the attribute *Hands Down*.

Besides the above, an analysis of inferential statistics can indicate other relationships and even confirm or reject initial data observations. The Wilcoxon Rank Sum test [33] is a non-parametric test for two populations with independent samples. With this test it may be possible to observe statistical similarities between a set of data.

Table 4 compares inferential tests among presentations from the same year at different times (e.g., comparison between the presentations 1 and 2 of year 1) and also presentations from different years at the same time (e.g., comparison between the first two presentations of years year 2 and year 1). Thus, different attributes among databases are compared in order to test the hypothesis in question. The right column in the table shows the total of different attributes for each comparison.

Comparing both years and by considering the presentations at the same time (temporal analysis), it is possible to observe that during the presentations, the differences in attributes have been reduced from 6 to 1. In comparison with the first presentation of the two years (year 1 and year 2), there were more statistically different attributes, with 6. It is important to note that the first presentations are the ones that contain more observations in the databases, which can generate some variation. Thus, for the first presentation, there were 6 statistically different attributes; for the second presentation, there were 3, and finally, for the last presentation, only 1. That may indicate that, in the end, students showed a more similar pattern with respect to the two years.

In general, these results obtained from the framework may indicate a trend of presentation at the end of each semester. The indicative is that, regarding the attributes, statistically, there is only one attribute that does not have a median indicating that it is similar in the final presentation of both years. In an educational context, this can be used in other ways, such as comparing different years (as in this study, but for more years), and also comparing different undergraduate courses. This can help to create better teaching methods, since this similarity or difference between presentations with a very similar context can be shown statistically.

### 5.3. Clustering

There are a few ways to segment observations in a database. These approaches return different results and assessments, which allows for a broad spectrum of visualization and understanding of the data. It can make student assessment more complete, and return a more granular way of “looking” at the data. In this article, two different clustering techniques were used, namely, the hierarchical approach and the k-means approach. Although other approaches can be employed, k-means is primarily one of the most widely used clustering techniques. The hierarchical analysis, on the other hand, allows us to visualize the formation of clusters and the outliers in the data. Both techniques can be complementary, and once used correctly, they can identify relevant attributes in the data.

#### 5.3.1. *k*-Means Clustering

Clustering by *k*-means, also known as Lloyd algorithm [34], is an iterative data partitioning algorithm that assigns *n* observations to exactly one cluster defined by a centroid. The *k*-value represents the number of clusters, and it is chosen before the algorithm starts. In many cases, it is difficult, and many times, it is not known how many clusters the problem should have. The algorithm that generates the *silhouettes* helps to visually assist in the identification of cases in which the k-means fails in some observations assigned to the clusters.

**Figure 5:**
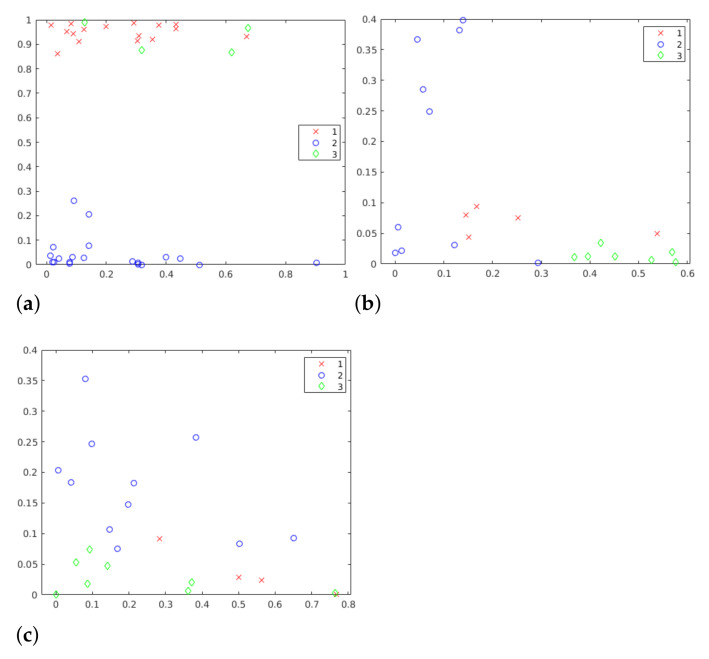
Scatter graphics in three different moments of year 1. (**a**) P1 year 1, *k* = 3, (**b**) P2 year 1, *k* = 3, (**c**) P3 year 1, *k* = 3.

The *k*-means algorithm was used in this framework as a way to segment students into different groups based on their presentation characteristics. To make this possible, 6 different *k* values were evaluated. Choosing *silhouettes* values can be challenging, but, in this work, it is possible to see in Figure 5 that 3 clusters is an appropriate value, since it presents homogeneous formation of clusters. Considering the centroids, it is possible to see three distinct patterns. It is important to highlight that the clusters formation depends on all attributes. Figure 5 illustrates only two of these attributes.

The use of these methods up to this point in the framework should return a very strong indicator for the number of clusters, whatever it may be. Thus, both silhouettes and k-means together allow us to identify the best number of clusters for the problem. Without a method for evaluating clusters, it is very difficult, and sometimes even impossible, to find the number of groups in a simple way, especially in exploratory studies where the expected results are unknown.

Once the centroids are generated, they must be evaluated to find information about the evolution and behavior of each cluster. Graphical visualizations can help a lot at this point. Polar, bar, or other charts can identify and highlight patterns. Still, a more granular data analysis, going through some observations in the databases, can also lead to the identification of patterns.

By analyzing the centroids, we can see which attributes are highlighted compared to others. This can be seen in the formation of some clusters, where some attributes separate the data into three precise “behaviors”. Still, in [35] are presented some attributes of excellent presenters during a presentation, and other attributes of poor performances. Poor presentations are related to withdrawn body language, for example, arms crossed. Good presentations include those related to open body postures, such as hand gestures and eye contact.

**Figure 6:**
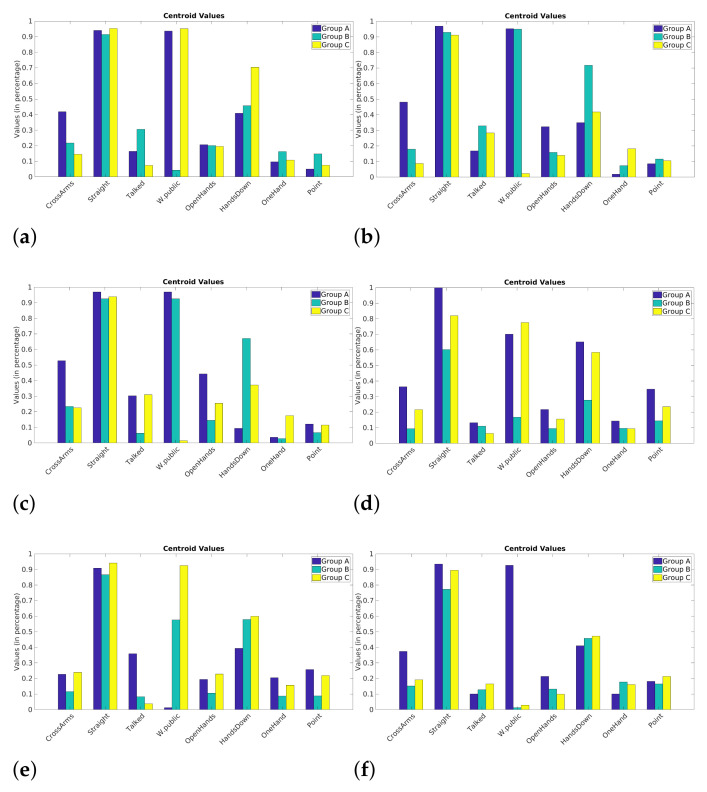
Bar charts of the centroids for each presentation. (**a**) P1 year 1, (**b**) P2 year 1, (**c**) P3 year 1, (**d**) P1 year 2, (**e**) P2 year 2, (**f**) P3 year 2.

In the year 1 presentations, it is possible to identify more homogeneously the three distinct clusters formed by k-means (see Figure 6). The *Cross Arms* attribute, for example, clearly separates into three patterns. Crossing the arms during presentations can be considered a withdrawn and less active attribute of behavior. It can be identified in the groups formed with higher values of this posture (*Cross Arms*), another one that has less and one that is still in between. Moreover, note that the attribute *Talked* also separates the data into three distinct behaviors. As expected, speech is a positive attribute as it indicates student interaction when presenting.

In general, the presentations in year 2 behave differently from the ones in year 1. As much as it is possible to separate into three distinct groups, the formation rule is not very clear, which makes comparisons with the year 1 difficult. It is possible to visualize that, initially, the behavior is somewhat random. Throughout the presentations (second and third presentation), more similar behavior is observed. Making the behavior more similar to the year 1, there is a group with a higher value in the attribute, another with a lower value and the third with an intermediate value.

After these analyses, it is clear that identifying similar patterns between the two years for this study was difficult. Knowing that, in the last presentations of each year, the attributes between those presentations are more similar (see Table 4), it is inferred that the centroids, and consequently the presentations, are more similar as well. As a result, the year 1 is somewhat more consistent in patterns. The variation between the three clusters was visual and simple to observe. Three distinct groups were found in the year 1 presentations. This process could help teachers and educators with an easy way to analyze student postures and presentations since these values (i.e., attribute values of each student during the presentation) are recorded. Still, even if it is not possible to trace a similarity connection between the two years (year 1 and year 2), as was the case in this study, teachers could identify what was done differently between classes, in order to improve the teaching process.

*K*-means proves to be a powerful tool in the characterization of students since the centroids indicate how the formation of clusters happens. The sum of results obtained so far by the framework can help students and educators to better understand the presentations in terms of postures and speech. The silhouettes help visually and analytically (average values) to find the number of groups present in the data. This can help teachers to segment students into smaller groups in order to identify the greatest difficulties for each of these groups.

#### 5.3.2. Hierarchical Clustering

**Figure 7:**
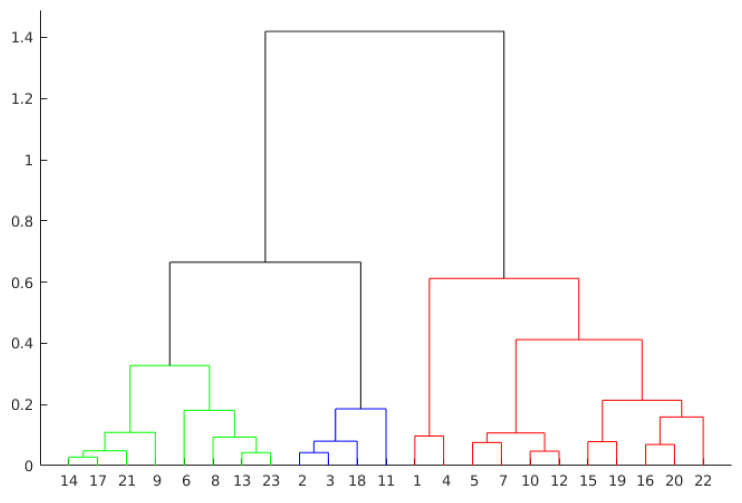
Dendrogram representation of presentation 3 in year 1.

**Figure 8:**
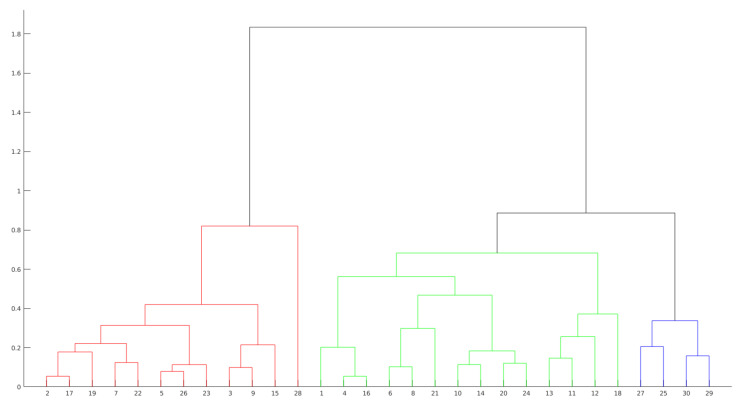
Dendrogram representation of presentation 3 in year 2.

Hierarchical Clustering is another approach to evaluate presentations by grouping observations. This technique creates the groups from the data in scale form, creating a tree that represents the clusters and the separation of the observations. This tree is not a single set of clusters, but a multilevel hierarchy, where clusters on one level are joined to the cluster on the next level. This relationship allows us to decide the level or scale to analyze the problem, since it illustrates the arrangement of the clusters produced by the corresponding analyses. As can be seen in Figure 7 and Figure 8, the colors clearly illustrate the number of ideal clusters, in this case, 3 for both years.

The hierarchical agglomerative clustering (HAC) requires to perform some previous steps (functions). First, it is measured the similarity between the objects in the database. Next, the objects are grouped in a hierarchical binary tree. Finally, we must decide where to cut the hierarchical tree to form the clusters. More formally, hierarchical clustering can be defined as follows. Given a set of *X* items to be grouped, and a similarity matrix XxX, the hierarchical clustering process [36] considers the following steps:

1. Assign each item to a different cluster, so for *X* items there will be *X* clusters;
2. Discover the closest pair of clusters and merge them into a single cluster;
3. Measure the distances between the new cluster and each of the old ones;
4. Repeat steps 2 and 3 until each item is grouped into a single *X*-size cluster.

Clustering algorithms differentiate observations using some measure of distance, with the inputs being the attributes of the problem. In this study, this information is the student’s positions and the speech attribute throughout the presentation. Given some attributes, it is possible to plot the observations on 3D graphics. Thus, by using different combinations of observations, it is possible to visualize the separation among them.

**Figure 9:**
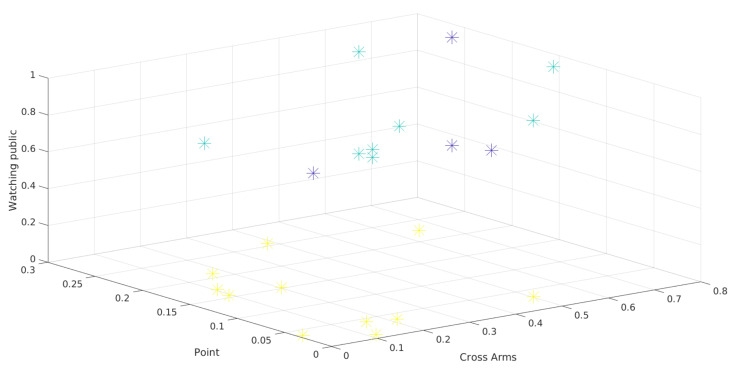
3D Scatter plot of presentation 3 in year 1.

**Figure 10:**
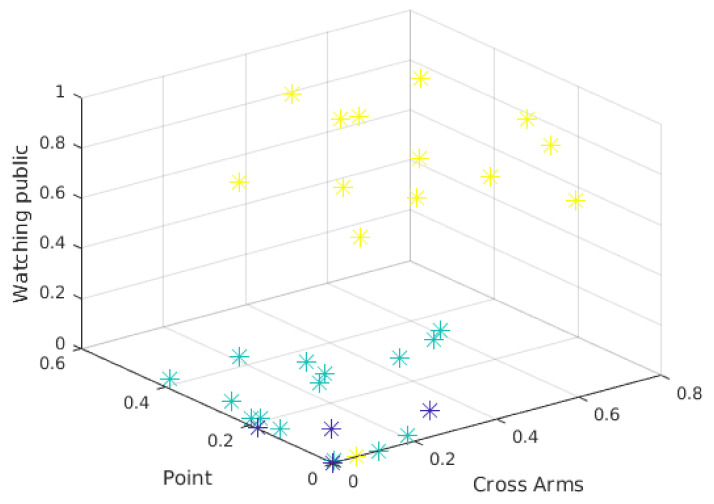
3D Scatter plot of presentation 3 in year 2.

Figure 9 and Figure 10 show the three-attribute 3D scatter plots for the third presentations of year 1 and year 2, respectively. Note that the chosen attributes (*Watching Public*, *Point*, and *Cross Arms*) allow the observations to be properly separated. It is important to say that the groups are not formed solely and exclusively by these three attributes. These are only the attributes being shown, so the separation between the clusters depends on other attributes.

The scatter plots and the clusters formed by the Hierarchical Clustering algorithm can help to overview the presentations. It can be difficult to identify attributes that separate the observations in the visualization. In fact, although Figure 9 and Figure 10 show a clear separation into two well-formed clusters, a third cluster with a smaller number of observations (the one represented in both figures by blue dots), can not be clearly separated from the others.

The 3D scatter plot can be useful when there are many attributes to be evaluated. A 2D scatter plot represents only 2 attributes, as the name suggests. Another attribute (3D) can be made easier to understand the behavior in the formation of clusters for a given clustering technique. With this graph, natural separations can also be visualized in the data based on 3 attributes. In the case of the present work, it can be seen that the attribute *watching public* is separating the observations. Whereas the other two attributes (*Cross Arms* and *Point*) do not separate the data as clearly.

The distances (heights) in the hierarchical tree (see Figure 7 and Figure 8) reflect the original distances of the data in an accurate form. Likewise, the investigation of natural divisions in the data that exist between the connections of the observations can be carried out. In a hierarchical tree, two objects from the original database are always linked at some level. The height of the connection represents the distance between two clusters that contains two observations. This height is known as the *cophenetic distance* [37] between the two objects. On the other hand, the horizontal connections between the observations represent the connections in the tree.

As can be seen in Figure 7, by dividing the groups in three clusters, the group in red presents the higher values. The other two groups are formed from a subdivision directly connected with the group in red. This behavior can also be seen in Figure 8, where two groups are formed from a previous subdivision linked with the group in red.

Otherwise, the analysis by agglomerative clustering can also show outliers in the data. Objects that do not connect hierarchically to groups can indicate students who do not fit into groups formed by the algorithm. Examples of this behavior are the observations 1 and 4 from the group highlighted in red in Figure 7.

### 5.4. Sequential Patterning Mining

The Sequential Pattern Mining (SPM) technique, first introduced by [38,39], provides a complementary perspective for the observation of the patterns as it allows one to see the postures in a chronological sequence, in different time points. SPM is a well-known technique in the field of data mining. In a sequential database, each sequence consists of a list of transactions [40]. Therefore, SPM allows finding all sequence patterns based on the *minimal support* initially provided by the user. The importance of applying sequential pattern mining in multimodal data was already previously explored in the literature by other authors (for instance in [41,42]). In the present work, presentations are evaluated individually, looking for the support of each of the sequences, that is, the number of occurrences of each sequence in the presentation. For each presentation of each year, we generate a set of more frequent sequences. The attributes used in the SPM algorithm are the same as the rest of the experiment (see Table 2).

The main goal of this stage is to identify the main sequences in the presentations, and if they occur in all the presentations throughout the year. It is hard to show all the sequence patterns found with the algorithm. As the databases have different attributes, the minimum support value varied between the analysis of both years. In this context, it was hard to analyze all the sequence patterns from year 1, since the experiments to identify a low *minimum support* returned large results.

The Sequential Pattern Mining Framework (SPMF) [43] is an open source data mining library. It offers more than 120 data mining algorithms implemented in JAVA. The tool also has a visualization screen to avoid programming efforts.

Initially, the data are sequences of *strings* representing attributes. For example, a sequence could be {*Hands Down*, *Hands Down*, *Hands Down*}, for a given observation. The SPMF software does not accept this type of input, so each attribute is associated to a numeric number. For instance, associating the *Hands Down* attribute to the number 1, the previous sequence becomes into {1 −1 1 −1 1 −1 −2 }. −1 indicates an interval between one item and another and −2 indicates the end of the observation sequence. To obtain the most frequent sequential patterns, the *PrefixSpan* algorithm [40], which is contained in SPMF, was used.

In general, in a first evaluation, the algorithm finds the main pattern, which contains the sequence of a single attribute, for example, *Cross Arms*. There are combinations in different quantities with this attribute, being a sequence of time that the student remains in that state. These patterns of a single attribute are not so relevant to the study, since the objective is to understand the student’s behavior, and it is not interesting to evaluate sequences where there are no changes of postures (attributes).

In our experiment, the algorithm returns many sequences ending in the *Hands Down* or the *Straight* attribute. Both attributes are common ways of being during a presentation: body position upright and with hands extended downwards, both at the end of the sequences.

In this study, it was decided to evaluate some specific sequences from the total found by the algorithm. These evaluated sequences are considered more relevant and in a way represent the general behavior of the other sequences present in the remaining results.

For the first presentation (see Table 5), some interesting sequences appear. For example, the first sequence in the table, with support equals 40. The support [44] of a sequence can be defined as the number of times that a sequence occurs in a sequential database. In that sequence, it is noted a more passive behavior, alternating in the position of crossed arms, low hands, and open hands (this may mean that the student is explaining something), and finishing in the *Straight* position. Furthermore, in many sequences, the *Cross Arms* attribute appears. This could happen because it is the first presentation, and the students still did not have enough presentation skills. It is noteworthy that the attribute *Hands Down* appears in several sequences, which is another indication that the student spends a lot of time in a passive attitude.

Other sequences of Table 5 have *Talked* as a relevant attribute. In the last sequence, it is noticed that the *Talked* attribute is followed by *One Hand*, which indicates that the student starts to point with one hand, trying to explain something after a period of verbal explanation.

The second presentation shows a similar behavior than the first one (see Table 6). Some variations of the attributes *Hands Down* and *Open Hands* in sequence with other attributes of a more passive characteristic. The attribute *One Hand* is highlighted in this presentation, with support 21. It appears in some sequences and particularly in the second one, where the students’ behavior includes the attributes *Hand on Face*, *Open Hands*, and at some point take the position of *One Hand* only and return to *Open Hands* again. This is an indication (indicated by the switching behavior for a hand) that the students explained something.

Here the *Point* attribute is highlighted. With support 21, it appears accompanied with the attribute *One Hand*. The *Talked* attribute shows an interesting behavior in the sequence with id 40 (see Table 6). It appears in the middle of other attributes at different times. It follows that the student explained something verbally and then lowered his hands, perhaps to explain something with both hands (non-verbal interaction), and then returned to the verbal explanation.

For the third presentation (see Table 7), the sequences maintain the pattern found in previous presentations. The *Cross Arms* attribute stands out again for its strong presence in the sequences in general. As in the other presentations, there are many attributes presenting sequences repeating same item many times, which were not exposed in the table for the reason explained above.

**Figure 11:**
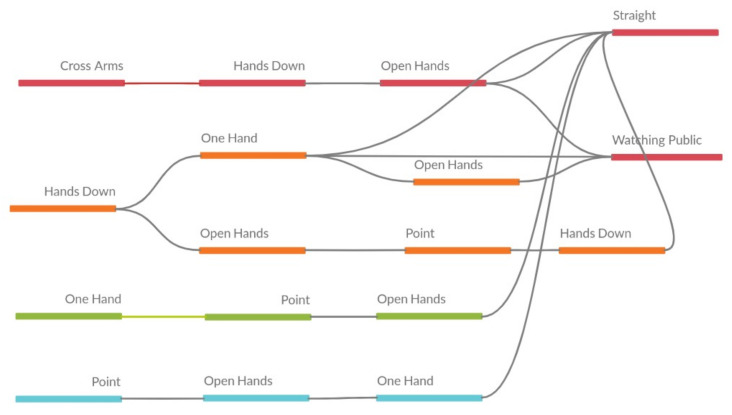
Visual representation of sequences from presentation 3 of year 1.

Evaluating presentations from these tables is difficult. The relationships between the attributes may not be directly displayed. Hence, Figure 11 presents another way to show the results. The relationships between the items in the sequences is presented directly, making it possible to visualize, for example, that many sequences finish in the *Straight* attribute (this behavior was observed without the visual help previously, but it is clearer with the figure).

Unlike the year 1 presentations, the year 2 ones show different sequences in general. The number of items in the sequences is smaller in year 2 than in year 1. It does not mean that there are no large sequences in the year 2 presentations. As in year 1, there are sequences with a single item repeatedly. In several sequences, the same item was found repeated eight times.

The attributes that appear most frequently in the year 1 presentations also appear in the year 2 presentations, for example, the attributes *Hands Down*, *One Hand*, *Straight* and *Open Hands*. As can be seen, this difference between the the presentations of both years can be explained by the statistical difference observed in Table 4. In the table, mainly between the first two presentations of each year, there are six statistically different attributes.

**Figure 12:**
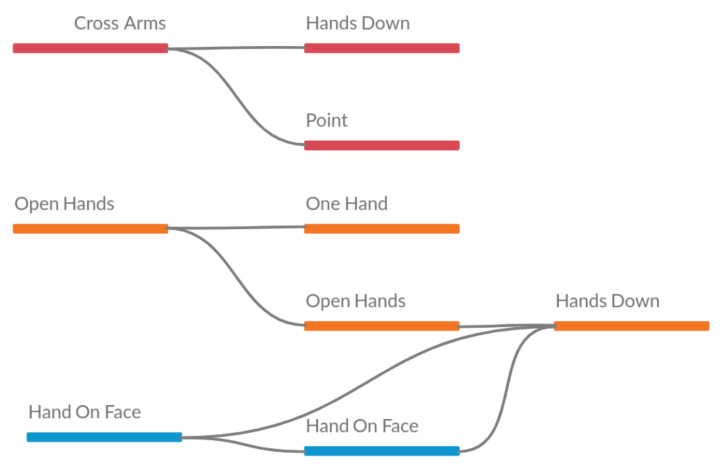
Visual representation of sequences from presentation 3 of year 2.

In the last presentation of year 2 (see Table 8), some patterns with the attribute *Cross Arms* are highlighted again. Similar pattern with the year 1 presentations, noting that at the end, according to the statistical data in the Table 4, the presentations should be more similar, since the number of different attributes is only 1. Figure 12 shows the visual representation of the table.

## 6. Discussion

The proposal of a conceptual framework for the analysis of body postures can guide the future work of other authors [15]. Also, it can provide a general understanding of the data used. At the same time that each process that is performed on the data returns a different result, adding knowledge and understanding of the problem studied.

The descriptive analysis of the data can clarify the problem about the evaluated attributes. The graphics that compare or analyze the evolution, with a temporal analysis, for example, the one used in Figure 4, can allow to identify relationships between the years, or a better understanding of a certain attribute during the period of student presentations. Trend charts allow to show common patterns between presentations from different years.

The inferential statistics analysis allows to find additional information for the study. As shown in Table 4, the *Wilcoxon rank sum* test allows to compare each presentation of each year at the same time, allowing to show that few attributes are statistically different. However, when comparing the two years just for the first presentation, half of the attributes are statistically different. Still, it can be observed that in the latest presentations there is only one statistically different attribute, representing a convergence. This indicates that, in the last presentations, students are presenting more similar in both years.

The k-means clustering algorithm allows identifying various attributes of the data. This algorithm is an important tool that segments data based on a mathematical model. With k-means, it is possible to identify the groups of behaviors (based on the attributes) generated by the students when presenting their work. The analysis of centroids (see Figure 6) allows a general understanding of these behaviors. However, the k-means algorithm alone becomes ineffective, since choosing the right *k*-number of clusters can be a difficult task. The *silhouettes* (see Figure 5) help both visually and numerically to find the value that better suits the problem.

Unsupervised machine learning algorithms can reveal different behaviors between presenters in oral presentation environments [45]. These methods can be relevant in exploratory data analysis. As shown, with k-means, formed groups “appear” with their different behaviors. With agglomerative clustering techniques, it is possible to observe the formation of groups and the connections between observations (presenters).

By using the *PrefixSpan* algorithm, the sequences show certain behaviors that, without the use of the algorithm, are difficult to visualize. For example, for the first sequence in presentation 3 of year 1 (see Table 7), it can be observed that the student is in a less active arms position during the whole sequence. What can be seen previously in the results of centroids (identification of different types of behaviors). This result can also be related to the results of descriptive statistics, the attributes that occur most, for example, *Straight*, also occur frequently in the sequences.

Analyzing presentations with multimodal data allows a better understanding of the learning process. It is difficult to estimate a complex mental state based solely on a behavioral attribute [18]. Through Kinect sensors, it is possible to collect data whose analysis allows us to identify different presentation patterns among the entire group (of students). Furthermore, in [18] is suggested that MMLA enables to detect some details that would be very difficult to perceive by human eyes. Attributes that previously would not have been possible or there would be no precision when observing the phenomenon, like the most frequent sequences of students.

For [46], MMLA still needs to be integrated into real-world environments, where educational environments are ecologically valid. In this sense, the integration of the presented framework with a standard Learning Management System (LMS) provide valuable information aiming at the individual assessment of the student, as well as of a class or classes in different years. Still, the analysis of sequences in a real environment, with more information such as teacher notes and impressions, could differentiate a good presentation from a bad one, since with this result one can observe the most frequent sequences in the presentations and track which students made which sequences.

The evaluation model created by the conceptual framework helps to reveal traits of the student’s behavior as they present it. With the evaluation of the centroid, it is also possible to identify patterns within the presentations (without comparing the years in the case study). At the end, going through all the steps, a database containing the time intervals that the student spends in each posture, must have as a result a student with well-defined presentation attributes. Also, if there are two or more years to be evaluated, it is possible to find the relationship (similarity between attributes) between the years. Thus, teachers and experts can compare and identify students presentation attributes to assign to learning gain or loss [47].

At last, it is important to highlight the framework was initially tested with data coming from a learning setting (students presentations) and more experimentation is needed to verify the generalization ability of the model. For that, it is necessary to collect more data from other scenarios. For instance, the framework could be applied as a complementary tool to revise and edit pitches strategies, adding an extra layer of evaluation to the discursive pattern observation [48].

## 7. Conclusions

This study present a framework using MMLA to assist the evaluation of complex environments from different sources. For example, when you compare presentations from different years, that is, year 1 and year 2, it is clearly possible to find out by inferential statistics test that the number of attributes decrease from 6 to 1. It means that, considering two semesters, students presents more homogeneous each other. It is also possible to see 3 different groups of behaviors in the presentations.

Some limitations were found when proposing the implementation of this framework with this data, especially in an attempt to compare the patterns created by the centroid when k=3. When evaluating the year 2, it was possible to observe a certain pattern of formation of the groups. Still, it is possible to evaluate the behavior during the 3 presentations of the year 1, but this was not possible for the year 2. The formation of the groups, due to the characteristics of the year 2 data, does not present a clear way of formation. This hindered the comparison between the years and did not allow an evolution in that direction. With this scenario, it was possible to observe that the comparison (between the years) by the framework, based on the students’ presentation characteristics can be a difficult task. It is important to note that inferential tests give another type of result and can be useful in that sense.

Another point identified as a limitation, natural in the aspect of the data, is the lack of information (other than body postures and speech). It would be important for the study, for example, the teacher’s notes or impressions to be present. This information could be used as a way to make other appointments. In the framework step of sequences, a view of presentation quality could be given more accurately. A classification or regression model could still be created if notes existed.

In this proposed framework it is also possible to evaluate the students’ presentations considering the *silhouettes* method and centroids. A visual method is also an important element in the evaluation of students’ presentations postures. In this case, the Microsoft Kinect sensors proves to be an excellent tool to clusters groups, and allows the comparisons of the presentations of the same year groups. For example, comparing the third and second presentations of year 1 did not show up any statistically different attribute.

The acquisition of other types of data, such as grades, or aspects of the students that can better characterize the student’s presentation can make the analyses more comprehensive. As mentioned, classifiers can be used, as well as the relationship between sequences and behavior can be better evaluated, since in this study this comparison was limited to the existing data.

At last, it is also required to carry out comprehensiveness analysis of distinct group of students from different areas of knowledge. It could be also considered a limitation of this work: the analysis of multiple students in the same area of knowledge. Furthermore, including the types of profiles should be integrated into a real time automatic classification of presentations software.

## Figures and Tables

**Table 1:** Details of the grouped framework processes.

Block	Process
A	Input: Student presentations. Activity: (1) Collect data from oral presentations; (2) Classify data in body/voice/action; (3) Tabulate the data; (4) Generate the database containing the observations with their times added to each attribute. Output: Database containing the students’ time intervals with respective body posture/voice.
B	Input: Database containing the students’ time intervals. Activity: (1) Visualize data comparing the databases; (2) Average the values of each attribute; (3) Inferential test comparing the two or more databases. Output: Information graphics; table comparing the most present attributes; Inferential comparison between databases and evaluated presentations.
C	Input: Database with observations. Activity: (1) Perform clustering techniques; (2) Save all clustering results for later evaluation, that is, index, centroids and so forth; (3) For each cluster value, run a cluster evaluation algorithm; (4) Visually evaluate the results if possible; (5) Create/use analytics methods to choose the best value of clusters to the problem; (6) Critical inspection on data results to find patterns. Output: Decision of the best cluster value for the problem; patterns that clustering method allows to find; plots showing the patterns and characterization of clusters.
D	Input: Database containing the students’ time intervals. Activity: (1) Transform the initial database into a sequential database (database formed by the sequence of the attributes for each student in each presentation; (2) Transform the data to the (default) format for the sequential pattern mining tool; (3) Test minimum support values to see what best fits the problem; (4) Create visual methods to discover the patterns; (5) Correlate the sequences results with the other data results. Output: More frequent sequences given a minimum support value; relation between the other results.

**Table 2:** Description of each database attribute.

Attribute	Description
Cross Arms	The presenter crossed both arms.
Downside	The presenter’s tilt is greater than 0.333, with −1 tilted back and 1 tilted forward.
Straight	The presenter’s tilt is between −0.333 and 0.333, with −1 tilted back and 1 tilted forward.
Watching Public	The presenter is looking at the audience.
Hand on Face	The presenter has one hand on the chin.
Open Hands	The presenter is explaining with both hands.
Hands Down	The presenter has his hands down.
One Hand	The presenter is explaining with one hand to down and the other doubled in an explanatory position.
Hand on Hip	The presenter has his hands on hip.
Hand on Head	The presenter has one hand on his head.
Point	The presenter is pointing with one hand.
Talked	The presenter’s voice is detected.

**Table 3:** Number of observations from each database.

Year	Presentation Period	Number of Students
Year 1	P1	40
	P2	22
	P3	22
Year 2	P1	59
	P2	45
	P3	34

**Table 4:** Inferential tests between databases.

Years	Presentations	Statistically Different Attributes
	P1XP2	1
1	P1XP3	0
	P2XP3	2
	P1XP2	3
Year 2	P1XP3	1
	P2XP3	0
	P1XP1	6
Year 1 × Year 2	P2XP2	3
	P3XP3	1

**Table 5:** Selected sequences from presentation 1 of year 1.

Id	Pattern	Sup
10	Cross Arms, Hands Down, Open Hands, Cross Arms, Straight	40
20	Cross Arms, Hands Down, Talked, Straight	39
30	Cross Arms, One Hand, Open Hands, Straight	39
40	Cross Arms, Talked, Straight	39
50	Hands Down, One Hand, Talked, Straight	39
60	Talked, Cross Arms, Hands Down	39
70	Cross Arms, Talked, One Hand, Straight	38
80	Hands Down, Talked, Cross Arms, Open Hands, Cross Arms, Hands Down	38
90	Talked, One Hand, Open Hands, Hands Down, Straight	38

**Table 6:** Selected sequences from presentation 2 of year 1.

Id	Pattern	Sup
10	Cross Arms, One Hand, Open Hands, Straight	21
20	Hand on Face, Hands Down, Open Hands, Hands Down, One Hand, Open Hands, Straight	21
30	Hands on Hip, Open Hands, Straight	21
40	One Hand, Open Hands, Talked, Open Hands, Talked, Straight	21
50	One Hand, Point, One Hand, Straight	21
60	Open Hands, One Hand, Point, Open Hands, Straight	21

**Table 7:** Selected sequences from presentation 3 of 2017.

Id	Pattern	Sup
10	Cross Arms, Hands Down, Open Hands, Straight	23
20	Cross Arms, Hands Down, Open Hands, Watching Public	23
30	Hands Down, One Hand, Straight	23
40	Hands Down, One Hand, Watching Public	23
50	One Hand, Point, Open Hands, Straight	23
60	Hands Down, Open Hands, Point, Hands Down, Straight	22
70	Hands Down, One Hand, Open Hands, Watching Public	22
80	Point, Open Hands, One Hand, Straight	22

**Table 8:** Selected sequences from presentation 3 of year 2.

Id	Pattern	Sup
10	Cross Arms, Hands Down	25
20	Hand on Face, Hands Down	25
30	Open Hands, One Hand	25
40	Open Hands, Open Hands, Hands Down	25
50	Hand on Face, Hand on Face, Hands Down	24
60	Cross Arms, Point	23

## Data Availability

The software developed is available in https://github.com/leikelen-team/Leikelen (accessed on 25 January 2021). The dataset analysed during the current study are available on reasonable request.
